# Immunotherapeutic interventions of Triple Negative Breast Cancer

**DOI:** 10.1186/s12967-018-1514-7

**Published:** 2018-05-30

**Authors:** Zehuan Li, Yiran Qiu, Weiqi Lu, Ying Jiang, Jin Wang

**Affiliations:** 1 Department of General Surgery, Zhongshan Hospital, Fudan University, 180 Fenglin Road, Xuhui District, Shanghai, 200032 People’s Republic of China; 2 Shanghai Public Health Clinical Center, Fudan University, 2901 Caolang Road, Jinshan District, Shanghai, 201508 People’s Republic of China

**Keywords:** Triple Negative Breast Cancer, Immunotherapy, Chemotherapy, Antibody therapies, Exosome

## Abstract

Triple Negative Breast Cancer (TNBC) is a highly heterogeneous subtype of breast cancer that lacks the expression of oestrogen receptors, progesterone receptors and human epidermal growth factor receptor 2. Although TNBC is sensitive to chemotherapy, the overall outcomes of TNBC are worse than for other breast cancers, and TNBC is still one of the most fatal diseases for women. With the discovery of antigens specifically expressed in TNBC cells and the developing technology of monoclonal antibodies, chimeric antigen receptors and cancer vaccines, immunotherapy is emerging as a novel promising option for TNBC. This review is mainly focused on the tumour microenvironment and host immunity, Triple Negative Breast Cancer and the clinical treatment of TNBC, novel therapies for cancer and immunotherapy for TNBC, and the future outlook for the treatment for TNBC and the interplay between the therapies, including immune checkpoint inhibitors, combination of immune checkpoint inhibitors with targeted treatments in TNBC, adoptive cell therapy, cancer vaccines. The review also highlights recent reports on the synergistic effects of immunotherapy and chemotherapy, antibody–drug conjugates, and exosomes, as potential multifunctional therapeutic agents in TNBC.

## Background

Tumours can be controlled by the immune system. This has been the subject of research for over a century, from the existence of tumour antigens and the cancer immunosurveillance hypothesis to the immunoediting hypothesis [1]. According to the cancer immunoediting hypothesis, tumour fate is shaped by the host immune system through three phases: the elimination, equilibrium and escape phases. The immune balance is first tilted to anti-tumour immunity in the elimination phase, and an intact and competent immune system detects and then destroys the developing tumour during immunosurveillance. Sporadic tumour cells may survive this editing phase and progress to the equilibrium phase, where the balance lies between anti-tumour and tumour-promoting factors, resulting in a functionally suppressed state of the tumour. Finally, the tumour cells acquire the ability to circumvent immune surveillance and destruction, and these immunologically sculpted tumours emerge with a progressively outgrowing status, establishing an immunosuppressive tumour microenvironment (TME) in the escape phase [1, 2].

It is not only infection-derived immunity, immune deregulation and autoimmunity preceding tumour development but also the intrinsic inflammation triggered by malignancies following tumour development that promotes cancer development and progression. As a result of these different forms of inflammation, the TME contains innate immune cells [macrophages, neutrophils, mast cells, myeloid-derived suppressor cells (MDSC), dendritic cells (DCs), and natural killer (NK) cells] and adaptive immune cells (T and B lymphocytes), in addition to the cancer cells and the surrounding stroma (fibroblasts, endothelial cells, pericytes, and mesenchymal cells) [3]. At the same time, inflammation also influences the host immune response to tumours and can be used in cancer immunotherapy and chemotherapy [3]. The immune response in tumours mainly relies on adaptive immunity, usually focusing on T cell-mediated cellular immunity [4]. CD8^+^ T cells evolve and kill tumour cells by excreting perforin, granzymes and IFN-γ [5]. There is evidence that some immune cells [DCs, MDSC, B cells, CD8^+^, CD4^+^ Th1, CD4^+^ Th17, CD4^+^ Tregs (regulatory T cells), macrophages, and neutrophils] exert both anti-tumourigenic and pro-tumourigenic effects and that others exert only pro-tumourigenic effects (mast cells, CD4^+^ Th2 cells) but that NK cells lack a protumourigenic effect [3]. DCs found in the TME play an important role in the induction of anti-tumour responses by cross-presenting antigens to CD4^+^ and CD8^+^ T cells [6]. While Tregs normally act against autoimmune diseases by suppressing self-reactive T cells, in the TME, they block anti-tumour responses by suppressing immune cells, such as CD8^+^ T cells, NK cells and DCs, and even participating in metastasis [7]. The depletion of Tregs in tumours by intratumoural NK cells, macrophages and neutrophils swings the immune balance towards a CD8^+^ T cell effector function, resulting in tumour suppression and regression [8]. Therefore, augmenting the anti-tumourigenic effect of CD8^+^ T cells, DCs and NK cells and minimizing the protumourigenic effect from Tregs may serve as potential immunotherapies similar to adoptive cell therapy (ACT). Moreover, the contents of the extracellular matrix (ECM), such as MMPs, prevalently change their activity and show an association with cancer progression and thus serve as potential immunotherapeutic targets [9]. Tumour antigens comprise tumour-associated antigens (TAA) and tumour-specific antigens (TSA), which can be used to specifically detect neoplasms [4]. These antigens, especially TSA, can be harnessed as candidates for tumour-specific antibody treatments, chimeric antigen receptor cell therapies or antibody–drug conjugates to accurately target tumours. Still, there are many sophisticated mechanisms that regulate this process, such as the autocrine effect of T cells, and we should concentrate on the aspect that is helpful to tumours by way of immunotherapy. Initial theories suggested that breast cancer (BC) is a non-immunogenic disease with fewer immunogenic tumour antigens [10], so BC has not been considered a cancer amenable to immunotherapeutic approaches for a long time; however, recent studies have shown evidence of significant immune cell infiltration of tumour-infiltrating lymphocytes (TILs) in a subset of patient tumours and a consolidated understanding that Triple Negative Breast Cancer (TNBC) is a highly heterogeneous breast cancer subtype, with higher expression levels of PD-L1 and more TILs. The TIL score can be a prognostic and predictive marker in standard therapies. High numbers of TILs correlate with increased pathological complete responses to neoadjuvant chemotherapy in TNBC, which demonstrates that the immune system plays an active role in the subgroup of breast cancer [11].

## Triple Negative Breast Cancer and clinical treatment for TNBC

Breast cancer is categorized into two types: non-invasive and invasive. Clinically, BC is also divided into three types: hormone receptor positive BC, human epidermal growth factor receptor 2 (HER2) positive BC and TNBC. As an invasive breast carcinoma, TNBC is defined by the absence of the three main breast cancer biomarkers, namely, the lack of the expression of oestrogen receptors (ERs), progesterone receptors (PRs) and HER2. According to its molecular heterogeneity, TNBC is divided into six subclasses, including basal-like (BL1 and BL2 of basal or myoepithelial origin), mesenchymal-like (M), mesenchymal stem-like (MSL), luminal androgen receptor expression (LAR), immunomodulatory (IM) and an unstable type [12]. Representing 10–20% of breast carcinomas, TNBC has a greater heterogeneity, for which specific therapies have not long been available, and it has a worse survival rate compared to other subtypes [13].

The lack of the identification of driver alterations that can be targeted, such as for traditional anti-Her2 therapy and endocrine therapy, leads to TNBC having a poorer prognosis than other invasive breast cancers [14]. Treatment options for TNBC are at the forefront of clinical research on breast cancer. Currently, TNBC patients undergo combination therapies, consisting of surgery, radiation, chemotherapy, newly developed targeted therapy and immunotherapy. The locoregional treatment of TNBC includes lumpectomy, breast-conserving surgery, total mastectomy and radiation therapy to the whole breast with or without a boost [15]. While some scientists maintain that TNBC requires an aggressive locoregional surgical option, necessarily removing all the breast tissue, emerging studies show that conservation therapy might improve locoregional outcome [16, 17]. Adjuvant/neoadjuvant chemotherapy is the mainstay of treatment for TNBC; the regimens include administering anthracyclines, taxanes, and/or platinum compounds, dose-dense AC (doxorubicin/cyclophosphamide) and TC (docetaxel/cyclophosphamide). Although TNBC is sensitive to chemotherapy, conventional chemotherapy options are far from satisfactory. However, the addition of platinum to standard chemotherapy could increase the proportion of patients achieving a pathologic complete response [18, 19]. Radiation therapy should follow chemotherapy when indicated; this is composed of whole breast radiation, chest wall radiation, regional nodal radiation and accelerated partial breast irradiation [20]. For years, TNBC was not typically considered as a cancer amenable for immunotherapy, until recent studies demonstrated several promising immunotherapeutic agents and the immune signature [21, 22]. TNBC is divided into five immune subtypes based on four distinct expression signatures, including T/B cell, interferon gamma (IFN-γ), transforming growth factor beta (TGF-β), and core-serum response, DCs, and/or macrophages (CSR), namely, T/B-cell/IFN high, IFN/CSR high, CSR high, TGFβ high, and immune low [22], which are the specific molecular signatures for the immune heterogeneity of TNBC and put immunotherapeutic interventions on the table for TNBC.

## Novel therapies for cancer and immunotherapy for TNBC

Currently, immunotherapy is emerging as an exciting treatment option for TNBC patients. In general, cancer immunotherapy encompasses immune checkpoint inhibitors and cytokines, adoptive cell therapy, and cancer vaccines.

## Immune checkpoint inhibitors

### Anti-PD-1 antibodies (Pembrolizumab, JS001, PDR001, and Nivolumab) and anti-PD-L1 antibodies (Atezolizumab and Durvalumab)

Programmed cell death protein 1 (PD-1) is an inhibitory immune checkpoint inhibitor that limits T-cell effector function within tissues, and it is expressed on the surfaces of immune effector cells, such as T-cells, B cells, NK cells, DCs, and many TILs [23], and has two known ligands, namely, PD-L1 and PD-L2 [24, 25]. PD-1 expression can be induced by tumour-derived IL-18 on immunosuppressive CD56 (dim) CD16 (dim)/-NK cells, and tumour-derived IL-18 is associated with a bad prognosis in patients with TNBC [26]. PD-1 can be activated mainly by PD-L1 (programmed cell death protein 1 ligand), which is expressed on T cells, B cells, NK cells, macrophages, DCs, epithelial cells, and vascular endothelial cells upon IFN-γ stimulation [27]. As critical inhibitory regulators, the PD-1/PD-L1 interactions in normal tissues can protect against tissue damage and limit inflammatory reactions mediated by T cells and other immune system components during infections [28]. Tumour cells also express PD-L1 and inhibit T cell responses by upregulating and binding PD-L1 to PD-1 on activated T cells, leading to immune exhaustion and downregulation of the local immune response [29]. Increased PD-L1 expression is seen on the surface of TNBC cells and has functional consequences on T cells, including decreasing their proliferation and increasing apoptosis, which provides the rationale for implementing therapeutic strategies targeting the PD-1/PD-L1 axis to unleash the effective killing of the TNBC cells [30–32].

Phase I studies evaluating antibodies targeting either PD-1 or PD-L1 show that these agents elicit durable, objective responses in patients with melanoma, NSCLC and renal-cell carcinoma [33, 34]. The first regulatory approved PD-1 inhibitor, Nivolumab, was launched for the treatment of unresectable melanoma in July 2014 [35]. With respect to TNBC, the Pembrolizumab, JS001, PDR001, and Nivolumab humanized anti-PD-1 monoclonal antibodies are currently being tested in multiple clinical trials. The anti-PD-L1 monoclonal antibodies Atezolizumab and Durvalumab are also yielding promising results [36]. The glycosylation of PD-L1 was recently found to be essential for the interaction of PD-L1/PD-1, and a newly developed antibody, STM108, which targets glycosylated PD-L1 (gPD-L1), can induce PD-L1 internalization to the lysosomes and degradation [37].

Although the anti-PD-1 and anti-PD-L1 mAbs have emerged as being of noteworthy significance in BC treatment, both were recently described as unsatisfactory as single therapeutic agents [38]. Tumour expression of PD-L1 may serve as a potential biomarker for clinical benefit. It was reported that high expression of either PD-1 or PD-L1 correlated with increased Foxp3^+^ Treg infiltration, and PD-1/PD-L1 and Tregs may work synergistically in immune evasion [39, 40]. Pembrolizumab did not affect the levels of Foxp3^+^ Tregs or change their phenotype or function but did block signalling via the PD1/PD-L1 axis in activated T cells [41]. Whether anti-PD-L1 mAb could affect Treg function remains unknown, but it might be a better choice than PD-1 blockade since PD-L1 is expressed on tumour cells while PD-1 is expressed on immune cells. However, not all PD-L1-expressing tumours respond to anti-PD-1/PD-L1 mAbs and it is noteworthy that some PD-L1-negative tumours such as PD-L1-negative lung cancer can respond to these agents [42]. To validate whether targeting PD-1 or PD-L1 is an option for treating TNBC, Pembrolizumab/Atezolizumab, used in neoadjuvant/adjuvant/metastatic settings in TNBC treatment, will be evaluated in ongoing clinical trials in Phase III studies (Table 1). Therefore, targeting PD-1 or PD-L1 may be an option for TNBC, and the efficacy of these immune checkpoint inhibitors remains to be improved.

**Table 1:** Ongoing clinical trials of immunotherapeutic interventions of TNBC

Targets/types	Drug	Patient population	Recruitment	Phase	ClinicalTrials. gov ID
Clinical trials in the neoadjuvant setting					
PD-1	Pembrolizumab	TNBC	Recruiting	II	NCT03145961
				III	NCT03036488
			Active, not recruiting	Ib	NCT02622074
			Not yet recruiting	II	NCT03289819
	PDR001	TNBC	Recruiting	II	NCT02938442
PD-L1	Atezolizumab	TNBC	Not yet recruiting	III	NCT03197935
			Recruiting	III	NCT03281954
				III	NCT02620280
				II	NCT02530489
	Durvalumab	TNBC	Recruiting	II	NCT02685059
				I/II	NCT02489448
PD-L1, PARP	Atezolizumab, Veliparib	TNBC, BRCA1/2 mutated, other BCs	Recruiting	II	NCT02849496
VEGF-A	Bevacizumab	TNBC	Recruiting	II	NCT02456857
EGFR	Panitumumab	TNBC	Recruiting	II	NCT02593175
		TN-IBC	Recruiting	II	NCT02876107
ACT	DC	BC	Recruiting	I/II	NCT03450044
	γδ T cells	BC	Recruiting	I/II	NCT03183206
	DC-CIK	BC	Active, not recruiting	II	NCT02491697
	ROR1 + CAR-T cells	TNBC, BCs, Leukemia, Lymphoma	Recruiting	I	NCT02706392
Vaccine	DC vaccine	TNBC and other BCs	Recruiting	I/II	NCT02018458
	Neoantigen DNA	TNBC	Recruiting	I	NCT03199040
	PVX-410	TNBC	Recruiting	I	NCT02826434
Clinical trials in the adjuvant setting					
PD-1	Pembrolizumab	TNBC	Recruiting	III	NCT03036488
		TNBC and other BCs	Recruiting	III	NCT02954874
PD-1, PARP	Pembrolizumab, Niraparib	TNBC, OC	Recruiting	I/II	NCT02657889
ACT	NK cells	BC	Recruiting	I/II	NCT02844335
	DC-CIK	TNBC, HCC, RCC, UBC, CRC, NSCLC	Recruiting	I/II	NCT02886897
Vaccine	PVX-410	TNBC	Recruiting	I	NCT02826434
Clinical trials in the metastatic setting					
PD-1	Pembrolizumab	TNBC	Active, not recruiting	III	NCT02555657
				II	NCT02447003
			Recruiting	III	NCT02819518
				II	NCT02768701
				II	NCT02755272
				I/II	NCT02734290
				Ib/II	NCT01676753
			Not yet recruiting	II	NCT03121352
		TNBC, IBC	Not yet recruiting	II	NCT03184558
	Nivolumab	TNBC	Recruiting	II	NCT03316586
				II	NCT02499367
	JS001	TNBC	Not yet recruiting	I	NCT03251313
			Recruiting	I	NCT03151447
	PDR001	TNBC, NSCLC, TC, Melanoma	Recruiting	Ib/II	NCT02404441
		TNBC, CRC, NSCLC	Recruiting	I	NCT02890069
PD-L1	Atezolizumab	TNBC	Not yet recruiting	II	NCT03164993
			Active, not recruiting	III	NCT02425891
			Recruiting	III	NCT03125902
				Ib/II	NCT02708680
				IIb	NCT01898117
	Durvalumab	TNBC	Recruiting	I/II	NCT02628132
	Avelumab	TNBC, SCCHN, SCLC, NSCLC, Melanoma,	Recruiting	Ib/II	NCT02554812
CTLA-4	Tremelimumab	TNBC, UBC, PDAC	Active, not recruiting	II	NCT02527434
PD-L1, CTLA-4	Durvalumab, Tremelimumab	TNBC, SCCHN, SCLC, GEJ, PDAC, ESCC	Recruiting	Ib	NCT02658214
PD-1, PARP	Pembrolizumab, Niraparib	TNBC, OC	Recruiting	I/II	NCT02657889
PD-L1, PARP	Durvalumab, Olaparib	TNBC	Recruiting	II	NCT03167619
	Durvalumab, Olaparib/Cediranib	TNBC, OC, CRC, NSCLC, SCLC, CRPC	Recruiting	I/II	NCT02484404
	Atezolizumab, Veliparib	TNBC, BRCA1/2 mutated, other BCs	Recruiting	II	NCT02849496
VEGF-A	Bevacizumab	TNBC and other BCs	Active, not recruiting	II	NCT00733408
ACT	NK cells	BC	Recruiting	I/II	NCT02843126
	Anti-MUC1 CAR-T cells	TNBC, HCC, NSCLC, PC	Recruiting	I/II	NCT02587689
	NKG2D CAR-T cells	TNBC, CRC, OC, UC, PC, MM	Recruiting	I/II	NCT03018405
Vaccine	haNK cell	TNBC	Not yet recruiting	Ib/II	NCT03387085
				Ib/II	NCT03175666
ADC	IMMU-132	TNBC	Recruiting	III	NCT02574455
			Not yet recruiting	II	NCT02161679
	CDX-011	TNBC	Active, not recruiting	II	NCT01997333
	SGN-LIV1A	TNBC	Recruiting	I	NCT01969643
ADC, PD-1	SGN-LIV1A, Pembrolizumab	TNBC	Recruiting	Ib/II	NCT03310957

### Anti-CTLA-4 antibodies (Ipilimumab and Tremelimumab)

Cytotoxic T lymphocyte-associated protein 4 (CTLA-4) is a T-cell inhibitory receptor that is expressed on activated CD8^+^ T cells and CD4^+^ regulatory T cells that express CD25 and Foxp3. CTLA-4, as a homologue of CD28, attenuates the T-cell immune response by binding to both CD80 (B7-1) and CD86 (B7-2) on DCs with affinities much greater than CD28, but the mechanism is unclear [43]. Therefore, CTLA-4 blockade probably removes inhibitory signals in the costimulatory pathway, resulting in the enhanced rejection of the tumour cells. The U.S. FDA approved Bristol-Myers Squibb’s anti-CTLA-4 treatment, called ipilimumab, for metastatic melanoma in 2011; ipilimumab showed a durable and potentially curative efficacy in potentiating tumour regression, with a higher complete response rate than in previous reports [44, 45].

High infiltration of Tregs in a tumour is usually associated with poor prognosis. Removal of Foxp3^+^ Tregs can evoke and enhance the anti-tumour immune response because they not only suppress aberrant immune responses but also inhibit anti-tumour immune responses [46]. The CTLA-4 antibody mediates anti-tumour immunity through Akt phosphorylation and the blockade of Foxp3^+^ Treg cells in the TME, which allows for potent T-cell expansion [47, 48]. A systematic review demonstrated that TNBC encompasses the highest incidence of TILs (20%; range, 4–37%) and the highest levels of Foxp3^+^ Tregs cells (70%; range, 65–76%) among breast cancer subtypes [49]. The elevated numbers of Foxp3^+^ Tregs (~ 66% of CD4^+^ T cells) may be therapeutic targets of CTLA-4 blockade antibodies in TNBC treatment [47]. While the disappearance of FoxP3^+^ Tregs is associated with pCR to neoadjuvant chemotherapy; how this could affect the response to anti-CTLA-4 treatment in TNBC remains controversial, with few investigations involved [50]. Limited preclinical data showed that anti-CTLA-4 did not significantly alter tumour growth except when Tregs were depleted [51]. While the anti-CTLA-4 antibodies Ipilimumab and Tremelimumab are currently undergoing clinical trials of TNBC (Table 1), more investigations are warranted to confirm the relationship between Treg infiltration and treatment response in TNBC.

## Combination of immune checkpoint inhibitors with targeted treatments in TNBC

Despite the remarkable benefits from the use of checkpoint inhibitors, clinical trials evaluating the use of combinations of checkpoint inhibitors to improve the response rate are now demonstrating that dual application of immune checkpoints inhibitors (blockade of CTLA-4 and PD-1/PD-L1 pathways) is a promising approach in TNBC. Additionally, combination therapy of checkpoint inhibitors with targeted treatments has shown the ability to increase the efficacy of immunotherapy and to slow down primary tumour outgrowth and metastasis, especially in the neoadjuvant background, and may simultaneously improve the tumour-specific T lymphocyte response, which is observed in multiple syngeneic TNBC models [38].

### Dual application of immune checkpoint inhibitors (anti-PD-1 and anti-CTLA-4) in TNBC

Although a number of trials with PD-1 inhibitors are encouraging for TNBC, only a fraction of treated patients detectably respond to this therapy. PD-1 and CTLA-4 exert their effects through distinct mechanisms [52], and people have gradually realized that simultaneously targeting both pathways may result in a synergistic effect on anti-tumour immunity, and the combination of the blockade of PD-1 and CTLA-4 possess more than twice the efficacy of either alone in melanoma and lung cancer [53–55]. Moreover, the blockade of both PD-1 and CTLA-4 restores T lymphocyte rejection function in tumours, especially when combined with GVAX vaccination (consisting of GM-CSF-expressing irradiated tumour cells) [56].

However, only few investigations involving BC were carried out. A combination of these two antibodies over comes tumour immunosuppression and effectively treats TNBC, with a regression of ~ 80% of tumours [47], allowing inactivated tumour-specific T lymphocytes to continue to expand and carry out effector functions, and this shifts the TME from suppressive to inflammatory [54]. Therefore, understanding of pharmacodynamic effects of the combination of these two antibodies in patients will definitely prompt the rational development of immune-based combinations against TNBC. Moreover, dual anti-PD-1 and anti-CTLA-4 combined with Cisplatin therapy not only led to an avid cytotoxic, rather than suppressive, immune response, characterized by enhanced DC activation, decreased FOXP3^+^ Tregs and concomitantly increased activation of CD8^+^CD4^+^ T cells (p < 0.05), but also more efficiently curtailed BRCA-1 deficient tumour growth (p = 0.008) [57].

### Anti-PD-1/PD-L1 mAbs combined with targeted therapies

#### Anti-PD-1/PD-L1 mAbs with EGFR inhibitors

As a member of the family of transmembrane receptors, EGFR (epidermal growth factor receptor) promotes cell proliferation and survival via initiating downstream signalling through the PI3K–AKT–mTOR and RAS–MEK pathways [58]. More importantly, EGFR works as a cotranscription factor that is localized in the nucleus and results in cancer progression. Currently, there are two main monoclonal antibodies (mAb) targeting EGFR, cetuximab and panitumumab. Cetuximab is a chimeric IgG1 mAb that blocks the ligand-induced phosphorylation of EGFR through binding to EGFR in cancer cells, with a higher affinity than both EGF and TGF-α [58]. In terms of breast cancer, nuclear EGFR expression is associated with resistance to gefitinib, an oral tyrosine kinase inhibitor (TKI), in vitro [59]. Moreover, nuclear EGFR expression also correlates with a more aggressive clinical behaviour in breast cancer [60]. EGFR expression is predominant in 89.5% of TNBC and can be transferred from TNBC cells to immune cells, leading to a decreased level of EGFR on TNBC cells, and its expression on immune cells correlates with a high tumour grade in TNBC patients (p = 0.02) [61, 62]. This indicates that EGFR can be transferred via trogocytosis from one cell to another at the time of contact and may alter the function of immune cells [63]. EGFR down-regulation also induces the reduction of PD-L1 expression on cancer cells [64], which could improve the efficacy of these inhibitors in TNBC. Further investigation is warranted to elucidate the mechanism of modulation of EGFR by immune cell contact to TNBC cells and its interaction with the reduction of PD-L1 expression, which may provide novel aspects for immunotherapy for TNBC.

Moreover, EGFR signalling may promote tumour growth and enhance immune escape by stimulating aerobic glycolysis in TNBC cells and producing lactate, which inhibits T cell activity [65]. In the past two decades, cetuximab has shown marked anti-tumour efficacy, including increasing the anti-tumour effects of doxorubicin [66], prolonging the PFS of TNBC patients in combination with cisplatin [67] and increasing the response rate for TNBC in combination with irinotecan [68]. Panitumumab is a fully humanized IgG G2 mAb, which is directed to EGFR to competitively inhibit EGFR binding, and has also been studied in TNBC [58]. Combined with chemotherapy, panitumumab also raises the pCR rate in TNBC patients [69]. In addition, dual EGFR inhibition, either by a combination of noncompetitive EGFR mAbs or a combination of an EGFR mAb and an EGFR tyrosine kinase inhibitor, might exert an improved anti-tumour effect in TNBC [70, 71]. Currently, a number of clinical trials have been carried out treating TNBC with anti-EGFR mAbs, and encouraging results will be yielded soon.

Despite the encouraging data on anti-PD-1/PD-L1, the efficacy of PD-1/PD-L1 inhibitors in patients with EGFR-activating mutations remains unclear. Preclinical investigations involving NSCLC and head and neck cancer revealed the immune modulatory effect of the EGFR signalling pathway, including repression of MHC I and MHC II [72], upregulation of PD-L1 expression (through the AKT/mTOR pathway, AKT-STAT3 pathway and ERK1/2 pathway) [73, 74], increasing the number and activity of immunosuppressive Tregs (through the EGFR/GSK-3b/Foxp3 axis) [75], and inhibiting the activity of CTLs [65]. Therefore, inhibiting EGFR by either mAbs or EGFR-TKIs could decrease the expression of PD-L1 in tumours.

However, the PD1/PD-L1 pathway might accounted for EGFR-TKIs resistance [76]. Delayed tumour growth and increased survival have been demonstrated in preclinical EGFR mutant lung cancer models treated with anti-PD-1 mAbs [77]. Together, combination of anti-PD-L1 mAbs with anti-EGFR mAbs or EGFR inhibitors will probably exert a synergetic effect. To better apply this combination to treatment of TNBC, further investigations are warranted to demonstrate the exact interaction between the PD-1/PD-L1 and EGFR pathways and the efficacy of this combination in TNBC.

#### Anti-PD-1/PD-L1 with VEGF inhibitors

Vascular endothelial growth factor A (VEGF-A) is released by tumour cells and is associated with tumour progression, angiogenesis and invasion in TNBC and other malignancies [78]. In a TNBC patient cohort, Su, Jung-Chen profiled VEGF-A levels and found associations with distant metastasis-free survival (DMFS) and disease-free survival (DFS), further demonstrating that high VEGF-A levels correlate with the risk of developing metastatic disease in TNBC [79]. Bevacizumab (BM) is a recombinant humanized mAb that blocks angiogenesis by targeting VEGF-A and was first approved for medical use in the United States in July 2004 [80]. Some maintain that the reduced number and function of tumour vessels induced by an antiangiogenic treatment might impact the intratumoural delivery of concurrently applied chemotherapy. Still, the efficacy of additional BM remains controversial across the world. Although the FDA ruled to withdraw its indication for advanced breast cancer in 2011, BM remains in application in other countries, including Australia. The addition of BM has been shown to improve the overall response rate (ORS) and PFS in patients with metastatic TNBC [81–83]. Although the randomized phase III BEATRICE trial showed no significant benefit from BM therapy for early TNBC [84], a trend towards an improved OS was observed in the triple-negative subgroup who received BM. Thus, BM not only helps to overcome adaptive resistance but also improves tumour perfusion to maintain intratumoural drug delivery when combined with a rational and complementary chemotherapy partner [85, 86]. The NSABPB-40 trials showed a significantly improved OS with BM [87], and the GINECO A-TaXel Phase 2 Study also showed high activity and manageable safety for the combination of paclitaxel, capecitabine and BM in TNBC [88].

As mentioned above, VEGF has an effect in immune regulation by suppressing antitumour immune responses [89]. VEGF-A produced in the TME enhances expression of PD-1 and other immune checkpoint molecules, and high levels of VEGF-A might be involved in resistance to PD-1 blockades, which could be reverted by targeting the VEGF-A/VEGFR pathway [90]. Blocking the VEGF pathway could also potentiate anti-PD-L1 mAb (atezolizumab) therapy and improve antigen-specific T-cell migration [91, 92]. A phase I study evaluating atezolizumab in combination with BM showed an overall response rate of 40% in metastatic renal cell carcinoma [92] and was well-tolerated in these patients without synergistic toxicity, which is important because both PD-1 and VEGF blockades were thought to cause unique adverse events, such as autoimmune diseases in some patients [91].

Bevacizumab has been shown to improve the ORS and PFS of patients with metastatic TNBC and improve intratumoural drug perfusion for combination chemotherapy (reviewed above), which shows that combining two agents may be a promising strategy. The similar synergistic anti-tumour effect in vivo can also be induced successfully by combining PD-1/PD-L1 and VEGF-A/VEGFR blockade [93], although few cases of patients with TNBC who had a significant response to combinational treatment of anti-VEGF with immune check-point blockade have been reported so far. In view of these, targeting VEGF-A/VEGFR could synergize with anti-PD-1/PD-L1 treatment and might be a good candidate for TNBC treatment.

#### Anti-PD-1/PD-L1 with PARP inhibitors

Similar to BRCA1-mutated tumours, 25% of sporadic breast cancers are deficient in DNA-repair, mainly in homologous recombination (HR) when double stranded DNA breakage (DSB) occurs [94]. PARP [Poly (ADP-ribose) polymerase] is a nuclear enzyme that participates in the repair of DNA single-strand breaks (SSBs) via the base excision repair pathway, and it is highly expressed in more than 90% of TNBC [95]. Inhibition of PARP results in accumulation of SSBs, which can lead to the formation of irreparable toxic DSBs in BRCA1/2 defective cells [96]. PARP inhibitors are promising agents for the treatment of BL-1 (basal-like 1) TNBC, which features an enriched cell cycle, elevated DNA damage response (ATR/BRCA), proliferation pathway, and cell-cycle checkpoint loss pathways [97]. PARP inhibitors serve as a group of novel oral anticancer drugs that are highly active in TNBC with selected mutations or epigenetic silencing of genes involved in the DNA damage response (DDR), including BRCA1 and BRCA2 [98]. However, PARP inhibitors can upregulate PD-L1 expression and enhance cancer-associated immunosuppression. Thus, anti-PD-1/PD-L1 mAbs may exert a supplementary and increased anti-tumour effect in combination with a PARP inhibitor [99]. To further confirm the efficacy of this combination, a few clinical trials have been carried out (Table 1).

#### Anti-PD-1/PD-L1 with anti-MMP-14 antibodies for potential application

The matrix metalloproteinase (MMP) family mediates ECM degradation and promotes cancer metastasis [100]. MMPs are localized to invadopodia, which are filamentous actin (F-actin)-rich cellular protrusions that degrade ECM, and MMP-14, a cell surface receptor that degrades collagen, is required for invadopodia formation, which activates secreted MMPs to promote cancer metastasis [101, 102]. Targeting the early steps in metastasis, such as ECM degradation and invasion in cancer cells, may improve outcomes in TNBC [103]. The enhanced expression of MMP-14 probably leads to concordantly enhanced metastasis in cancer models and is associated with a comparatively poor prognosis in human breast cancer [104]. Several selective MMP-14 antibodies have been exploited. DX-2400, a potent and highly selective human antibody inhibitor of MMP-14 activity, significantly decreases MMP-14 activity, decreases immunosuppressive TGF-β, polarizes macrophages to an anti-tumour phenotype, and increases iNOS, leading to impaired primary tumour growth and an improved response to radiation therapy [105]. Moreover, some specific scFv antibodies bind outside the catalytic cleft of MMP-14 and impactfully prevent its proteolytic functions at the surface of cells [106]. Fab R2C7 is another inhibitory Fab with an excellent selectivity for MMP-14 [107]. Fab 3369 inhibits MMP-14-mediated ECM degradation and MDA-MB-231 cell invasion. Through an analysis of lung tissue sections from mice using a human TNBC xenograft model randomized between control IgG and IgG 3369 treatment groups, the MMP-14 inhibitory antibody 3369 was found to limit MDA-MB-231 tumour xenograft growth and metastasis [108]. More interestingly, Binbing Ling also demonstrated the potential of MMP-14 blockade to disrupt the immunosuppressive TME in metastatic breast cancers, while a number of immune regulatory genes were altered with MMP-14 blockade [108]. However, at this point, there have been no reports or clinical trials on targeting MMP-14 together with anti-PD-1/PD-L1 treatment; more potential therapies can be applied in patients with TNBC in the future.

**Fig. 1:**
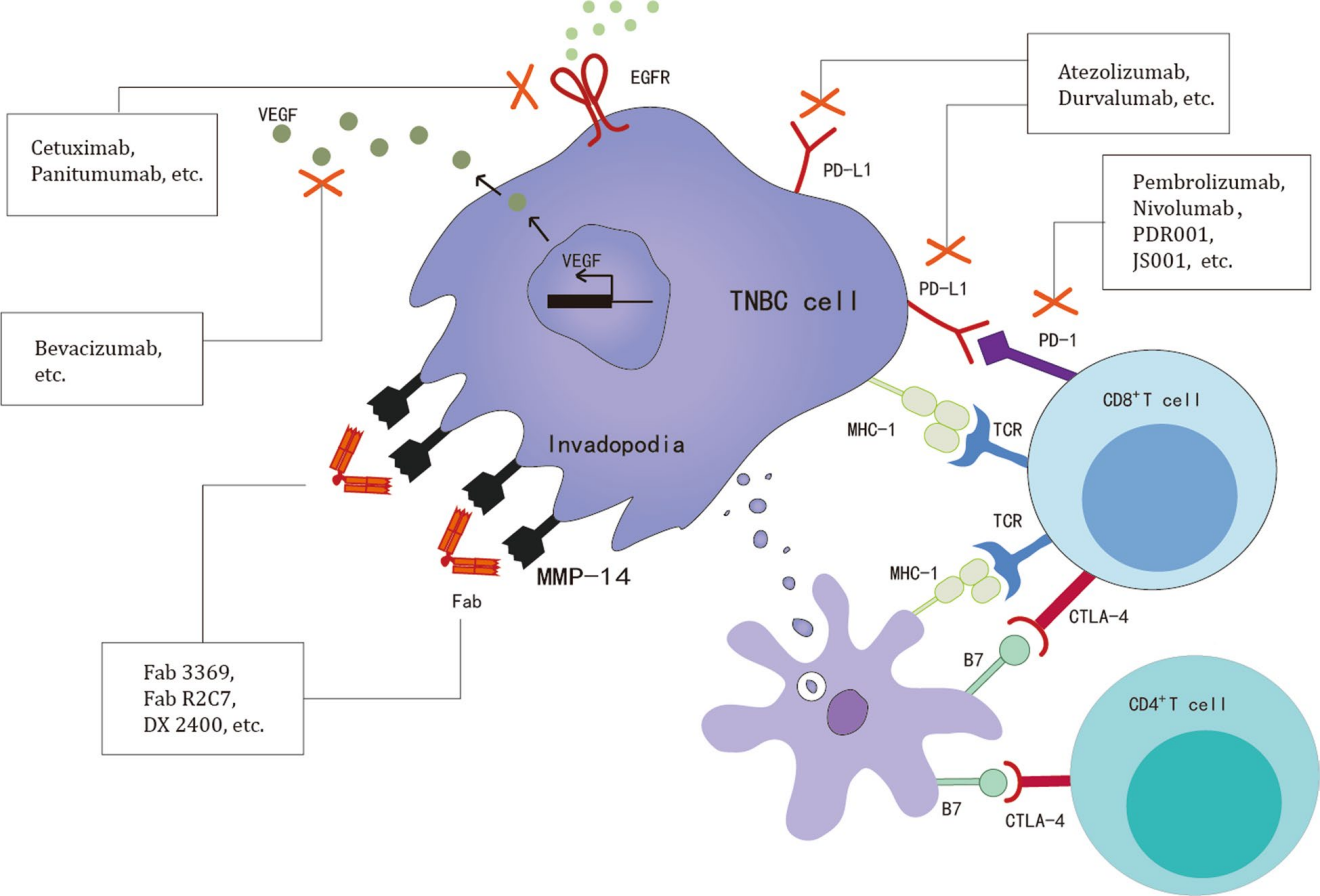
Current and potential future immune-related drug targets in TNBC, including immune checkpoint inhibitors, cytokines, and their antibodies

Currently, a number of clinical trials involving anti-immune checkpoint inhibitors, cytokines and their antibodies (anti-PD-1: Pembrolizumab, JS001, PDR001, Nivolumab; anti-PD-L1: Atezolizumab, Durvalumab; anti-CTLA-4: Ipilimumab, Tremelimumab; anti-EGFR mAb: Cetuximab, Panitumumab; anti-VEGF-A mAb: Bevacizumab; anti-MMP-14 antibody: Fab 3369, Fab R2C7, DX 2400) have been carried out to treat TNBC (Fig. 1). These agents might have a notably broad range of action with consequent problems influencing the future applications in the treatment of TNBC. Therefore, more research on specified immune checkpoint inhibitors and monoclonal antibodies for TNBC is urgently required.

## Adoptive cell therapy (ACT)

Adoptive cell therapy is a promising and likely potent approach to inducing anti-tumour immune responses via the isolation of highly active and tumour-specific lymphocytes, including TILs, cytotoxic T lymphocytes (CTLs), Th cells, NK and DC cells, large-scale ex vivo expansion and the activation of these lymphocytes for autologous therapy [109, 110]. As the ultimate effector cells, CTLs express a unique T-cell-antigen receptor (TCR) that confers specificity for the particular target antigen. The productive engagement of TCR/MHC/antigen complexes on the target-cell surface triggers the CTL’s effector functions and induces the destruction of the target cell through the release of inflammatory cytokines, including tumour-necrosis factor-α (TNF-α), IFN-γ, FAS ligand (FASL), TNF-related apoptosis-inducing ligand (TRAIL) and cytotoxic degranulation [109]. NK cells play a critical role in cancer immunosurveillance. Therapies utilizing NK cells have shown great promise due to advances in NK cell expansion protocols [111, 112], which are classified as follows: (1) harnessing endogenous responses by NK stimulants or targeting agents and (2) using exogenous NK cells via haematopoietic stem cell transplant (HSCT) or ACT [113]. Clinical studies show that NK cells from BC patients can be expanded and have a high cytotoxic ability to kill breast cancer cells [113, 114]. Until now, ACT has been successfully used for the treatment of patients with metastatic melanoma, leukaemia, and neuroblastoma [115, 116]. Therefore, with host immune environment manipulation, including the pre-administration host immunosuppression and concurrent cytokine administration, with the transferred cells, more novel adoptive cell therapies can be applied in patients with TNBC in the future.

### Chimeric antigen receptors T-cell-based therapy

Having underdone almost 30 years of evolution since the first generation of chimeric antigen receptors (CARs) was developed in 1989 by Gross G [117], this technology is still in its early stage of exploitation and development and facing nonignorable challenges, namely, the inability to control the rate of cytokine release and tumour lysis. In 2010, Rosenberg published chimeric antigen receptor therapy (CAR therapy)—a personalized treatment involving genetically modifying a patient’s T cells to make them target tumour cells [118]. Currently, multiple organizations are developing CARs against a host of targets, from epidermal growth factor receptor variant III (EGFRvIII) and mesothelin to CD19, CD20, CD30, CD33, and CD138 [119]. To date, ACT therapy has gained much attention in the past decade, especially CAR-T cell therapy, which grafts an arbitrary specificity onto an immune effector T cell. CARs are fusion receptors that are composed of an antibody-derived single-chain variable fragment (scFv) coupled via hinge and transmembrane elements to a T cell signalling and co-stimulatory domain. Recently, the novel CAR-T was engineered to express interleukin-7 (IL-7) and CCL19 [120].

A few agents have been developed and applied to CAR-T cells for TNBC treatment. Several CAR-T based ACTs were used in current clinical trials, such as ROR1^+^ CAR-T cells, anti-MUC1 CAR-T cells and NKG2D CAR-T cells. More candidates are in development. A patented antibody (TAB 004) specifically recognizes a tumour-associated form of MUC1 (tMUC1) in > 90% of human TNBC, and the antigenic isoform recognized by TAB 004 is completely hidden in normal epithelia, which makes it extremely safe for the development of CAR-T cells [121]. Mesothelin might have promise as a unique tumour associated antigen for TNBC; it has been screened in 99 primary breast cancers (it was overexpressed in 67% TNBC but < 5% ER(+) or Her2-neu^+^ type, and was undetectable in non-neoplastic mammary epithelium) [122]. TEM8 CAR-T cells were also recently postulated as a promising CAR-T-cell-based therapy, in which the TEM8 CAR T cells induce the regression of both established, localized patient-derived xenograft tumours (PDX) and lung metastatic TNBC cell line-derived xenograft tumours, by both killing the TEM8^+^ TNBC tumour cells and targeting the tumour endothelium to block tumour neovascularization [123]. Moreover, there is an increasing number of potential targets that are comparably highly expressed in TNBCs, such as FRα and brachyury, that have been developed [124, 125], which may provide implications for clinical tumour antigen screening for CAR-T cell-based therapies.

### T cell receptors (TCRs)—engineered T cells

TCR-engineered T cells are CD8^+^ T cells efficiently engineered to express TCRs recognizing intracellular antigens processed by major histocompatibility (MHC) proteins, which can target and kill cancer cells expressing appropriate antigens [126]. Investigations of TCR-engineered T cells began over two decades ago, with a number of preclinical studies showing their ability to mediate tumour lysis and eradication. The large amount of attention has recently led to the increased development of this ACT, which has shown encouraging outcomes in studies of TCR-engineered T cells directed against NY-ESO-1, MAGE and GP100, with significant clinical successes in patients with colorectal carcinoma, synovial sarcoma, metastatic melanoma and multiple myeloma [126, 127]. Recently, placenta-specific 1 (PLAC1)-specific HLA-A0201-restricted TCR-engineered CD8^+^ T cells were developed to kill breast cancer cells by producing IFN-γ and TNF-α [128]. However, more widespread utilization of TCR-engineered T cells in solid tumours such as TNBC calls for the enhancement of long-term survival and function of the cells, as well as closed culture procedures capable of expanding T cells to sufficient numbers for clinical application. Fortunately, modular systems and semi-automated devices have been developed and used in large-scale manufacturing [129]. Moreover, CRISPR/Cas9 technology can improve the function and sensitivity of TCR-engineered T cells to antigens by redirecting primary T cells with a pan-cancer reactive γδ TCR in combination with endogenous TCR-β knockout [130]. Of note, TCR-engineered T cells expressing a high level of PD-1 could reduce their functional activity; the efficacy of these cells may be augmented when used in combination with anti-PD-1 mAbs [131, 132].

### Tumour-infiltrating lymphocytes

Tumour-infiltrating lymphocytes are white blood cells that have left the bloodstream and migrated into a tumour; they are composed of a mix of different types of mononuclear immune cells (T cells, B cells, NK cells, and macrophages) in variable proportions, of which T cells are dominant [133]. Known as indicators of immunogenicity, TILs are not only remarkably prognostic but are also significantly predictive for tumours in cutaneous melanoma [134], colorectal cancer [135, 136], urothelial carcinoma [137] and breast cancer [133]. TILs also show a robust prognostic and predictive value in TNBC, indicating the immune status of the tumour and determining the efficacy of conventional chemotherapy [138, 139]. However, paradoxical results have emerged in several studies, suggesting that some CD8^+^ T cell-infiltrated tumours show poor outcome. Some efficient CD8^+^ T cell invasion and infiltration in the tumour is correlated with good outcome, while some patients show poor outcome due to the accumulation of CD8^+^ T cells in the tumour-associated stroma, with poor infiltration in the tumour epithelium [140]. A high amount of TILs suggests an immune status of a tumour near an equilibrium between then cancer and immune equilibrium in TNBC [141]. Surgical resection of the primary tumour has also been found to tilt the balance towards the immune system and, therefore, result in a better prognosis of high TILs in TNBC [141]. Low TILs correlate with a greater clonal heterogeneity and mutation load in TNBC, which may consequently lead to tumour escape from immune surveillance. It is not TILs alone, however, but also the balance between distinct immune components in the TME that impacts the outcome of patients with TNBC. TNBC patients experiencing tumour recurrence show a decreased content of TILs and an increased number of CD163^+^ tumour-associated macrophages (TAMs) compared with those without recurrence [142]. High levels of tumour infiltrating CD8^+^ T cells may reflect an improved prognosis with chemotherapy sensitivity, and TAMs correlate with a poor outcome in TNBC patients [138]. On the other hand, the heterogeneity of the CD8^+^ T cell distribution also crucially composes the newly identified distinct immune microenvironment of TNBC [140].

Despite the tremendous progress made regarding ACT, the potential of ACT therapy is facing inevitable challenges, such as expanding specific cells, including CD8^+^ T cells. Increasing efforts have been made to expand CD8^+^ T cells, NK cells and DCs, which will probably be utilized in autologous ACT for TNBC. Expanded NK cells from PBMCs isolated from breast cancer patients survive in vivo and prevent the establishment and growth of TNBC cells in a xenograft mouse model [114]. Cytokine-induced killer (CIK) cells have also emerged as a potential ACT option, for their impressive efficacy in improving DFS and OS in TNBC patients [143]. Further improvements in ACT therapy call for a deeper understanding of the immunological processes, the ability of lymphocytes to persist in vivo and to travel to tumours, unexpected toxicities to normal tissue, and the mechanisms of ACT augmentation by previous host immunosuppression.

## Cancer vaccine

Sipuleucel-T, a personalized treatment working by programming each patient’s immune system, is a therapeutic vaccine for prostate cancer approved by the FDA in April 2010, showing an ability to improve overall survival in patients with castrate-resistant prostate cancer. The MAGE-3 protein-based vaccine is also undergoing phase III clinical trial testing in patients with melanoma and NSCLC [144]. To date, a tremendous number of cancer vaccines, from peptide vaccines such as PPV, to DNA vaccines such as the hDR5 DNA vaccine, and from cytokine vaccines such as combined GM-CSF to Lymphocyte vaccines such as a DC-related vaccine, are carving the way for TNBC treatment.

### Cancer-testis antigens (CTA) as a vaccine target

Cancer-testis antigens are a heterogeneous group of TAAs displaying the ideal characteristics of promising immunotherapeutic targets [145]. Several CTAs are specifically expressed in TNBC, including SP17, NY-ESO-1 and MAGE group [146]. Sperm Protein 17 (SP17) was originally identified in the flagellum of rabbit spermatozoa [147] and is localized in the human fibrous sheath (FS) of sperm flagellum during different phases of spermatozoa maturation [148]. SP17-specific cytotoxic T lymphocytes were successfully generated from normal donors [149]. SP17 is aberrantly expressed in ovarian cancers [150], oesophageal cancer [151], nervous system tumours [152], endometrial and cervical cancers [153], NSCLC [154], and myeloma [155], and is associated with the migratory and motility capacity of tumour cells, indicating a link between the gene expression patterns in germinal and tumour cells of different histological origins [156] and is suggested as a promising target for immunotherapy. SP17 is expressed in both breast cancer cell lines and primary breast tumours and, importantly, in the TNBC subtype. Moreover, the detected specific anti-SP17 antibodies in patient sera was used to generate SP17-specific, HLA class I-restricted, cytotoxic T lymphocytes capable of efficiently killing breast cancer cells [145]. Early clinical data and assays in some respects support the rationale for further investigations of SP17 for tumour vaccines [157]. NY-ESO-1 expression is an independent good prognostic factor (p = 0.046) in TNBC and leads to a high humoural immune response associated with higher TILs [158, 159]. Therefore, the detection of NY-ESO-1 expression in TNBC might be useful for selecting patients who may benefit from cancer vaccination therapy.

### Personalized peptide vaccination (PPV)

A novel regimen of personalized peptide vaccination, which was developed by Itoh K, has been used in a phase II trial [160], and selected vaccine antigens from a pool of 31 peptides showed boosted immune activation and a noted clinical response [160]. An intramuscular vaccination with TNF-related apoptosis-inducing ligand receptor TRAIL R2 or death receptor 5 (DR5) DNA, as a novel promising vaccine target, not only elicits proapoptotic antibodies and IFN-γ-producing T cells (p < 0.001) but also inhibits TNBC SUM159 growth by hDR5 immune serum (p = 0.02) [161]. GM-CSF, combined with breast cancer stem cell- associated antigens and cytosine-phosphorothioate-guanine oligodeoxynucleotides (CpG-ODNs) in spontaneous breast cancer TA2 mice, is efficacious not only in suppressing tumour growth (p = 0.035) but also in activating and accumulating CD3^+^CD8^+^ T cells to kill tumour cells (p = 0.001) (P < 0.05) [162].

### Antigen-presenting cell (APC) and DC-based tumour vaccination

APC and DC-based tumour vaccination have been deeply investigated and met with noted success in several malignancies, including TNBC. O’Shaughnessy successfully gave 10 TNBC patients autologous monocyte-derived DC vaccinations intratumourally and subcutaneously during preop chemotherapy, which turned out to be safe [163]. Day-3 DCs fused with whole apoptotic breast cancer MDA-MB-231 cells could elicit effective specific anti-tumour T cell responses and might be developed as a prospective vaccine for BC immunotherapy [164]. Co-cultured DCs isolated from healthy donors and transduced by Runx2 with T cells also induce CTL and kill TNBC cells [165].

In summary, TNBC is regarded as the prime subtype of breast cancers and is amenable to immune checkpoint inhibition. There are currently a number of selective inhibitors, such as immune checkpoint inhibitors, cytokines, and their antibodies, as mentioned above, and adoptive cell therapy and cancer vaccines have also gained much attention in the past decade. Recently, some of these targets have entered into initiated clinical trials of immunotherapeutic interventions of TNBC (Table 1), which may have a notably broad range of action, with consequent problems influencing the future applications in the treatment of TNBC.

## Efficacy of immunotherapy and future perspectives in TNBC

### Synergistic effect of immunotherapy and chemotherapy

Increasing number of studies suggest that the chemotherapeutic agents such as anthracyclines, Cisplatin and Carboplatin exert their anti-tumour activity not only by directing cytotoxic effects but also by changing the TIL distribution. Chemotherapy with anthracyclines also requires priming of IFN-γ producing CD8^+^ T cells in mice [166].

There are several studies involving combinations among immune checkpoint blockade, anti-EGFR antibody, ACT, Cisplatin, Carboplatin, Cyclophosphamide, Doxisome, and Paclitaxel, and the efficacy of chemotherapy requires immune cells, such as CD8^+^ cells, and cytokines, such as IFN-γ, genes, such as CD8α/β, IFN-γ, IL-1β and IL-17, and the IL-1β/IL-1R signalling pathway [167]. There are several mechanistic reasons that this combination works. First, chemotherapy alters the immune gene signatures in TNBC and several metabolic pathways are also upregulated in response to cytotoxic therapy [168]. Second, both chemotherapy and checkpoint antibodies induce positive changes in the TME and improve the outcome of TNBC patients. High levels of IFN-γ, TIL counts, and the corresponding enhanced immune response are associated with better clinical responses to chemotherapy and a high proportion of pCR [11, 57, 167, 169]. Although some chemotherapies may harm lymphocytes, such as CD4^+^, CD20^+^ and CD68^+^ cells, they decrease immunosuppressive Foxp3^+^ Tregs, maintain or even increase CD8^+^ effectors, and invert the CD4/CD8 ratio [50, 170]. Finally, chemotherapy-induced cells release ATP to activate the NLRP3 inflammasome in DCs by releasing IL-1β [166]. Therefore, these changeable gene signatures in TNBC and the immune cells and cytokines of TME are maybe the reason why chemotherapy improves immunotherapy; although, it could possibly harm lymphocytes.

On the other hand, TNBC responses to anti-PD-1 or anti-PD-L1 are modest (< 20%) and a high expression of PD-L1 is associated with an enhanced response, which demonstrates that immune checkpoint blockade in the neoadjuvant setting enhances the effects of the conventional neoadjuvant chemotherapy alone [169]. Treatment with anti-PD-1 during DC maturation enhances DC survival [171]. The synergistic therapeutic activity of Doxisome (liposomal encapsulated formulation of Doxorubicin) with anti-PD1 is due to increased DCs infiltration in the TME, which internalizes tumour antigens, induces T cell anti-tumour immune responses, and increases the therapy response in TNBC [172]. Chemotherapies, containing anti-EGFR/VEGF mAbs, also show significantly positive outcomes [68, 87, 88]. A small sample size trial shows that cyclophosphamide, thiotepa and carboplatin, as first-line regimens, combined with DC-CIK immunotherapy and followed by oral low dosage cyclophosphamide, as maintenance therapy, were effective and safe for metastatic TNBC exposure compared to the previously used anthracyclines and taxane-based adjuvant chemotherapy [173]. All these studies demonstrated that the association between the immune response and the clinical outcomes of TNBC probably correlate with the role of the immune cells in the administration of cytotoxic chemotherapy.

### Antibody–drug conjugates (ADC)

Antibody–drug conjugates are a burgeoning new treatment modality that utilizes monoclonal antibodies that recognize TAAs/TSAs and preferably internalizes when bound to the tumour cells to deliver highly potent cytotoxic agents [174]. There are at least 100 clinical trials involving ADCs in different types of cancers, including melanoma, gastrointestinal cancer, pancreatic cancer, colorectal cancer, ovarian cancer, cervical cancer, and endometrial cancer. (https://clinicaltrials.gov/ct2/results?cond=antibody±drug±conjugate&term=&cntry=&state=&city=&dist=), and three ADCs (SGN-LIV1A, glembatumumab vedotin (CDX-011, CR011-vcMMAE) and Sacituzumab Govitecan (MMU-132, hRS7-SN-38) are carried out to treat TNBC among these clinical trials.

The zinc transporter LIV-1 (SLC39A6) is over-expressed in TNBC and is maintained after hormonal therapy in primary and metastatic sites. SGN-LIV1A is an anti-LIV-1 antibody linked to a potent microtube disrupting agent monomethyl auristatin E (MMAE) via a cleavable dipeptide linker and displays specific cytotoxicity both in vitro and in vivo against LIV-1 expressing cancer cells by internalizing and trafficking to the lysosome [175]. IMMU-132 is made from a humanized anti-Trop-2 (expressed in TNBC) mAb (hRS7) conjugated with SN-38 (the active metabolite of irinotecan) and is well tolerated and induces early and durable responses in heavily pretreated patients with metastatic TNBC, which mediates early pro-apoptosis signalling events (p53 and p21 WAF1/Cip1) and leads to the cleavage of PARP [176, 177]. A combination of IMMU-132 plus PARP inhibitors, such as olaparib or talazoparib, produces significantly improved anti-tumour effects and delays tumour progression compared with monotherapy in mice bearing BRCA1/2-mutated TNBC [178]. Glycoprotein NMB (gpNMB) is a novel type I transmembrane protein that is overexpressed in most breast cancers and promotes metastases by mediating intercellular adhesion, promoting tissue repair, regulating cell growth and differentiation, and down-modulating anti-tumour T-cell responses. CDX-011 is composed of an anti-gpNMB mAb and MMAE and has a clinically acceptable safety profile in its first study in breast cancer, showing 12 weeks of PFS in 60% of TNBC patients treated with CDX-011 [179]. The EMERGE trial also confirmed its enhanced activity in patients with gpNMB-overexpressing TNBC [180]. More pivotal clinical trials concerning these promising ADCs are currently underway and more new ADCs, such as protein tyrosine kinase 7 (PTK7)-targeted ADC, and novel potential candidates for ADC, such as STM108, are in development [37, 181].

### Exosomes as potential multifunctional therapeutic agents in TNBC

Exosomes, known as small 30–100 nm sized extracellular vesicles, are present in many and perhaps all eukaryotic fluids regardless of whether they are normal or malignant, and are particles that encapsulate contents, such as microRNAs. Exosome messaging contributes to TME interactions, including immune suppression and immune escape, invasive growth, adhesion, angiogenesis, radiation resistance, chemo-resistance and genetic intercellular exchange, and can manipulate tumour progression and metastatic cascade [182, 183]. Exosomes were recently reported to play dramatically positive roles in the clinic as targets, biomarkers or even therapeutic agents. Exosomes can be isolated from the blood of patients with various malignancies through various methods, including chemical binding, immunoaffinity capture and differential ultracentrifugation and serve as biomarkers for diagnosis and the monitoring of tumour progression [184]. The innovation of cancer exome-based diagnostic routines would potentiate a route towards the development of personalized immunotherapies [144].

**Fig. 2:**
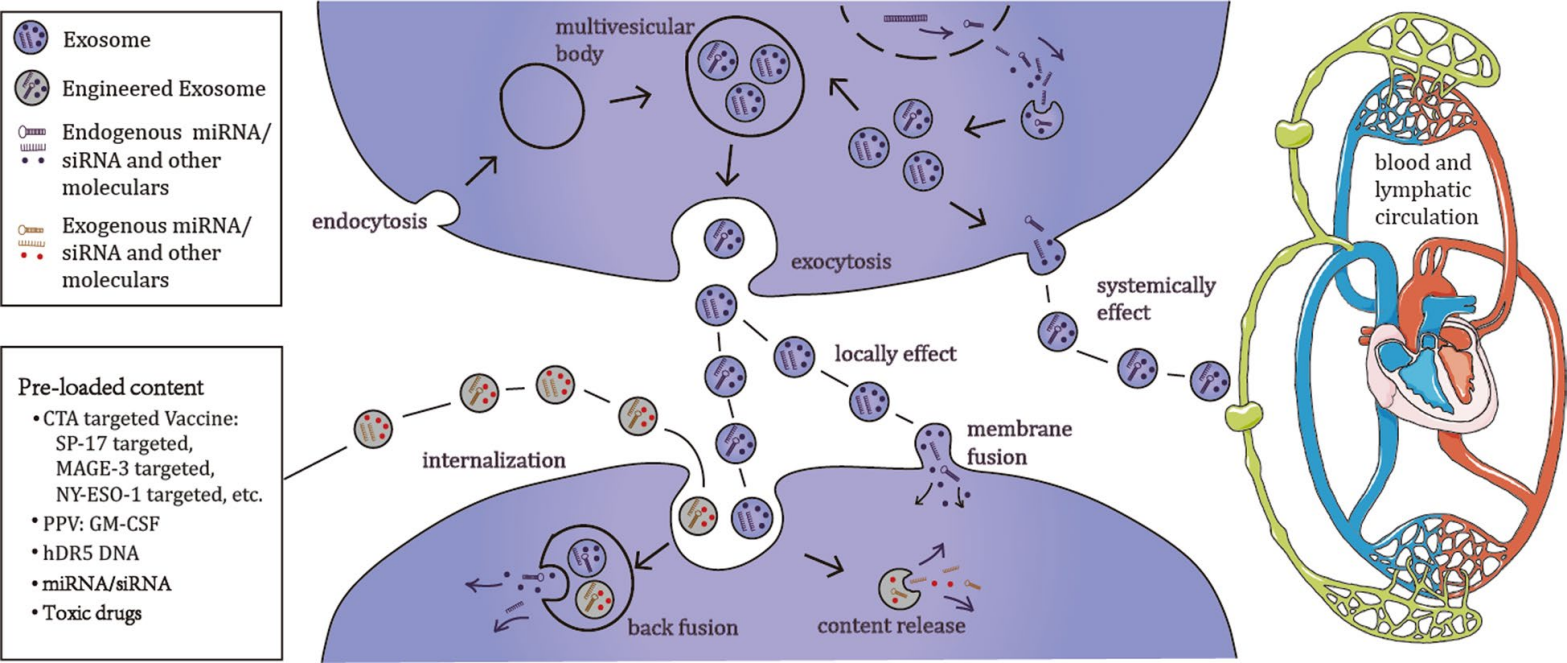
Exosomes may serve as a therapeutic option for TNBC. The formation and secretion of exosomes and various types of cancer vaccine therapies and potential vaccine targets for TNBC

Finally, there is possibility of developing exosomes as therapeutic agents for TNBC. Moreover, some TNBC cells possessing exosome-mediated apoptosis-inducing activity have been investigated [185]. Exosomes that are released from either normal cells or breast cancer cells can either locally or systemically effect neighbouring cells, travelling through the blood and/or lymphoid nodes from other tissues, which is probably regulated by extracellular exosomes [186, 187]. Antigens may increase immune stimulatory capacities when they are carried by exosomes, which could optimize the application of CAR-T therapy and will improve the engineered exosomes, which are emerging as novel vehicles for cancer vaccine development by APC technology. Exosome-based vaccines and exosome pre-loaded miRNAs/siRNAs/toxic drugs will be therapeutic options for TNBC [188, 189] (Fig. 2). Although exosome-secretion could eliminate some cytotoxic drugs such as doxorubicin and cisplatin, modified exosomes may serve as a novel drug delivery system offering the transportation of anti-cancer drugs with a lower immunogenicity and toxicity [190, 191]. Therefore, the mechanism of this interaction and whether engineered exosomes could be used to inhibit TNBC progression requires further elucidation.

## Conclusions

Overall, it is evident from these studies that immunotherapy is emerging as a novel promising option for TNBC, and there is the possibility of developing exosomes as potential multifunctional therapeutic agents for TNBC. In the future, modern therapeutics needs hold a huge guarantee for the development of affordable, novel and safe antibodies, antibody–drug conjugates, adoptive cell therapies, and cancer vaccines. We expect that exosomes from the tumour microenvironment are likely to become the most effective vaccines for TNBC. With the promising outcomes of immunotherapies, several immunotherapies are being evaluated in phase III trials, which means there would be more therapeutic choices, other than chemotherapy, for TNBC. Immunotherapeutic interventions in TNBC possibly exert a noteworthy effect based on the ongoing clinical trials in the neoadjuvant setting, adjuvant setting and metastatic setting. The synergistic effects and safety concerns of immunotherapy and chemotherapy need be addressed. As it moves towards phase III trials in this field, with a growing interest from pharmaceutical agencies, the final treatment pattern of Triple Negative Breast Cancer will be determined by these ongoing clinical trials, which will lead to a more refined immunotherapy. However, in-depth scientific investigations are required to completely determine the safety and effectiveness of these immunotherapies and open new avenues for the better management of patients with TNBC.

## Abbreviations

AC: doxorubicin/cyclophosphamide
ADC: antibody–drug conjugates
APC: antigen-presenting cell
AT: docetaxel/cyclophosphamide
BC: breast cancer
BM: bevacizumab
CAR: chimeric antigen receptors
CDX-011: glembatumumab vedotin
CIK cell: cytokine-induced killer
CpG-ODNs: cytosine-phosphorothioate-guanine oligodeoxynucleotides
CRC: colorectal cancer
CRPC: castrate-resistant prostate cancer
CTA: cancer testis antigen
CTLA-4: cytotoxic T-lymphocyte-associated protein 4
CTLs: cytotoxic T lymphocytes
DC: dendritic cell
DFS: disease-free survival
EGFRvIII: epidermal growth factor receptor variant III
ERs: oestrogen receptors
GEJ: gastric/gastro-oesophageal junction
GM-CSF: granulocyte-macrophage colony-stimulating factor
gpNMB: glycoprotein NMB
haNK: high-affinity Natural Killer
HCC: Hepatocellular Carcinoma
HER2: human epidermal growth factor receptor 2
HNC: head and neck cancer
IFN-γ: interferon gamma
IMMU-132: Sacituzumab Govitecan
IL-7: interleukin-7
mAb: monoclonal antibodies
MM: multiple myeloma
MMAE: monomethyl auristatin E
MMP: matrix metalloproteinase
MSLN: mesothelin
NK cell: natural killer cell
NSCLC: non-small cell lung cancer
OC: ovarian cancer
PBMC: peripheral blood mononuclear cells
PC: prostate cancer
PD-1: programmed cell death protein 1
PD-L1: programmed death-ligand 1
PDAC: pancreatic ductal adenocarcinoma
PPV: personalized peptide vaccination
PRs: progesterone receptors
PTK7: protein tyrosine kinase 7
RCC: renal cell carcinoma
ROR1^+^: receptor tyrosine kinase-like orphan receptor 1 positive
SCCHN: squamous cell carcinoma of the head and neck
SCLC: small cell lung cancer
SP17: Sperm Protein 17
TAA: tumour-associated antigens
TC: thyroid cancer
TCR: T-cell-antigen receptor
TGFβ: growth factor beta
TKI: tyrosine kinase inhibitor
TME: tumour microenvironment
TNBC: Triple-Negative Breast Cancer
TNF-α: tumour-necrosis factor-α
TRAIL: TNF-related apoptosis-inducing ligand
TNIBC: Triple-Negative Inflammatory Breast Cancer
Tregs: the regulatory T cells
TSA: tumour-specific antigens
UBC: urothelial bladder cancer
UC: urothelial carcinoma
